# Professionals’ Perceptions: “Why is Lead Poisoning Prevalent in Lancaster County?”

**DOI:** 10.3390/ijerph16132281

**Published:** 2019-06-27

**Authors:** Harriet Okatch, Margaret Cherney, Brittany Mokshefsky, Madeline Kuon, Sarah Scheuring, Emily Ritchey, Jiayi Chen

**Affiliations:** Franklin and Marshall College, Lancaster, PA 17603, USA

**Keywords:** lead poisoning, in-depth interviews, knowledge levels, housing status, competing interests, Lancaster, professionals, children

## Abstract

Background: The prevalence of lead poisoning in children under the age of six years living in Lancaster County, Pennsylvania continues to be greater than the state-wide prevalence for this age group. This study aims to determine the factors that contribute to the high lead poisoning rates. Methods: For this qualitative study, the researchers recruited a convenience sample of professionals providing healthcare and social welfare services in the county. Researchers conducted in-depth interviews with participants. The research team audio recorded, transcribed verbatim, and analyzed each interview using NVivo 12 software. Results: The 16 interviewed participants identified factors that contribute to high lead poisoning rates including knowledge levels, housing status, and competing interests. Specifically, low knowledge levels, renting as opposed to home ownership, and having competing interests seemed to minimize the attention directed towards preventing lead poisoning. The experts offered recommendations to address the high lead poisoning rates including increasing lead knowledge levels of both community members and landlords, through creating and distributing health promotion material, enacting policies to empower renters, and systematically collaborating to streamline lead poisoning related information and services. Conclusions: Findings provide insights to factors that Lancaster can address to achieve a decrease in lead poisoning rates. This study provides information that can be utilized by public health professionals to develop appropriate interventions.

## 1. Introduction

Nationwide, almost half a million children between the ages of one and five years have elevated blood lead levels (EBLL); a blood lead level greater than 5 ug/dL is considered the actionable level, as defined by the Centers for Disease Control and Prevention [1]. As early as 1956, studies have shown that, in children, exposure to lead can impact growth and development [2]. Prior research shows that EBLLs have been associated with harmful effects in children as they grow, such as neurobehavioral deficits including hyperactivity, withdrawal, development delays [3], decreased cognitive function, correlated lower IQ scores, and behavioral issues [4,5,6]. A study by Stretesky and Lynch showed that lead poisoning can affect one’s hormonal system, resulting in aggressive, violent behavior, or even homicide [7].

In response to these outcomes, professionals have made various efforts to provide a solution. At the national and state level, policy-makers are continuously setting laws to prevent children from encountering lead. Since 1992, landlords are required to disclose any known information on lead-based hazards to potential tenants before they sign a leasing agreement [8]. In June of 2018, Pennsylvania passed a bill amendment requiring all publicly funded schools to test their water for lead and release the results to the public, in the event of elevated levels [9].

At the local level, in Lancaster County, Pennsylvania, there are a number of organizations that focus on raising awareness of the danger lead poses and distribute health promotion materials to educate the community on exposures, effects, and prevention of lead poisoning. These organizations also provide information about available resources to help reduce lead levels. These organizations include the Lancaster Lead Coalition, the Partnership for Public Health, and the UPMC Pinnacle Lead Poisoning Prevention and Education Program. In addition, elementary schools in Lancaster annually test their water for lead. Of the 12 elementary schools in the School District of Lancaster, only two had lead levels higher than 0.01 mg/L of lead [10]. Social services programs have also begun to provide information about the threat of lead and the benefits of lead screening for children. In 2016, both Lancaster County and City were recipients of a USD1.33M grant from the Department of Housing and Urban Development (HUD) [11] (p. 6). HUD distributes grants to state and local health departments for lead abatement in homes, built before 1978 when lead-based paint was banned.

Despite the efforts, the rate of lead poisoning in Lancaster continues to remain high. The 2015 Lead Surveillance Annual Report indicated that 6.81%of children under the age of six years in Lancaster had EBLLs compared to 4.62% in the state of Pennsylvania, and the rates for 2017 were 7.50% in the county compared to 4.52% in the state [12,13]. Therefore, this study aimed to identify the factors that have contributed to the persistent high prevalence of EBLLs in children under the age of six years in Lancaster County. The study investigated the perspectives of both professionals/stakeholders and community members. However, this article explores the perceptions of the professionals and stakeholders and their beliefs of the factors affecting elevated blood lead levels in children and identifies the main themes that emerged from the interviews. 

## 2. Materials and Methods

### 2.1. Setting

This study was based in Lancaster County; a county in the state of Pennsylvania. As of July 2018, Lancaster had a population to 543,557 and 6.5% of the population are children under the age of five years. Of this population 4.9% are foreign-born. Almost 85% of Lancaster County residents have a high school education, 26.5% have greater than a high school diploma, and 15.2% have less than a high school diploma. The median household income for Lancaster is $61,492 and 9.9% of the population live in poverty. Almost 69% of housing units are owner occupied and the median rent is $957. Of those under age 65 years, 12.0% are without health insurance [14].

### 2.2. Design

The research team selected a qualitative methodology to determine the factors that contribute to high lead poisoning rates in Lancaster. This study design allowed for deep and rich exploration of the participants’ opinions and perspectives. Based on existing literature and the lead poisoning profile of Lancaster, the principal investigator (HO) developed an interview guide. The guide consisted of two sections: the first focused on collecting demographic information (age, sex, race/ethnicity, and occupation) and the second contained five open-ended questions. The principal investigator designed these questions to gain an understanding of the professionals’ perspectives on the Lancaster community’s knowledge levels of lead and its associated risks, their understanding of the policies related to lead remediation in Lancaster County homes, and recommendations to reduce lead poisoning rates in Lancaster County. All researchers were trained in the methodology of conducting semistructured interviews, including strategies to remain impartial and ask probing questions, when the situation warranted.

### 2.3. Recruitment and Sampling

The study team sent a recruitment letter to all the members of the Lancaster Lead Coalition. Initially, stakeholders who were part of the Lancaster Lead Coalition were purposively selected (*N* = 6). The study adopted a snow-ball sampling technique, based on recommendations from the Lancaster Lead Coalition member respondents (*N* = 10). A total of 16 professionals were included in the study. For the purposes of this study, professionals were defined as individuals who currently work directly or indirectly with families with a child under the age of six providing healthcare or welfare services (e.g., nurse practitioners) and/or in a field in which they are knowledgeable about the lead poisoning issues in Lancaster County (e.g., academics).

### 2.4. Data Collection

Researchers conducted in-depth interviews with all consenting participants. At least two researchers conducted each interview; one researcher acted as the main interviewer and asked the protocol questions while the second researcher recorded high level summary notes. Contingent upon consent from the respondent, researchers recorded each interview to ensure accuracy of response transcripts. However, note taking was a precautionary step in the case of technological malfunctions. The note taker(s) also asked follow-up questions, when necessary. Researchers traveled to conduct interviews with individual stakeholders in academic and professional settings. Each interview lasted 30–45 minutes. All interviews took place between November 2017 and April 2018.

### 2.5. Data Analysis

Researchers transcribed all interview audio recordings verbatim and entered transcripts into NVivo 12 software (QSR International, Melbourne, Australia). Using two transcripts, the seven-person research team collectively participated in code book development. The code book consisted of twenty codes including: education, testing, exposure, regulations, landlords, prevention, symptoms, and remediation. Two members of the research team (MK and HO) coded subsequent transcripts. The coding went through multiple iterations, resulting in eight codes: recommendations, landlords, education, regulations, testing, prevention, exposure, and limitations. The kappa coefficient, an NVivo 12 feature, allowed the researchers to assess intercoder reliability. The coders achieved near perfect agreement with a Kappa score of 0.88 (range: 0.84–0.99).

### 2.6. Ethical Considerations

The study was approved by the Institutional Review Board of Franklin and Marshall College (Code: #R_xo4vZpN8x1u0hJD). All participants gave consent to be part of the study by signing a consent form detailing the purpose of the study and the potential benefits and risks. 

## 3. Results

Researchers coded transcripts from in-depth interviews with 16 participants and identified eight themes. The participants were nurse practitioners, program managers, health officers, grant administrators, lead inspectors, or professors. Program managers worked in roles in which they could potentially make decisions and/or implement programs to address lead poisoning within their organizations. Participants were 32–73 years with an average of 53 years; their average number of years of work experience was 16 years. 

The researchers identified the three main themes, on the basis of frequency, that professionals acknowledged as contributing to the high lead poisoning rates in Lancaster; (i) low levels of knowledge about lead poisoning, (ii) housing status, and (iii) competing interests. Within the responses the professionals provided suggestions for addressing each of these factors to minimize lead poisoning. The following paragraphs summarize the findings and provide verbatim quotes from the interviews. 

### 3.1. Low Levels of Knowledge of Lead Poisoning

When asked how educated they felt the community was about lead poisoning, all of the interviewees evaluated the knowledge levels to be low, and/or increasing. Respondents noted that knowledge levels about lead and lead poisoning were increasing, among both community members and professionals, including physicians. Respondents also described knowledge levels as variable within the community, with some sectors of the community exhibiting higher knowledge levels than others. 

“I think that there are isolated individuals who may have become aware of lead and its toxicity because their child has had an elevated lead blood level, they know someone who’s had a problem. But I think, in general, the population really lacks awareness of lead and its toxicity, and I think it has to do with a few things, like environmental health literacy in general, science literacy in general, and the awareness that there are things that are parts of our everyday life that can really impact us.”

“I think there are some folks who are extremely well-versed in it and knowledgeable. I think there are some people that know about it but don’t think it affects them, and I think there’s some people that are oblivious, so I think it really runs a gamut.”

Respondents identified people who are more knowledgeable as those who have had experience with lead poisoning of a family member, relative, or friend. Participants indicated that the lead contaminated water crisis in Flint, Michigan helped increase awareness about lead, though to a limited extent.

“I think there was lead on the radar from everything that transpired in Flint and there was good coverage that LNP (local newspaper) did around Lancaster’s elevated levels, but I am not sure that that has become common knowledge, that Lancaster has as big of a problem as we do.”

Participants shared some observations of active efforts to educate community members about lead and lead poisoning and also shared recommendations about how to increase knowledge levels on lead poisoning. Participants universally stated that preparing, or adopting brochures, and distributing them would be an effective way to increase awareness. However, one participant added that the distribution of health promotion materials had to be at an appropriate reading level to attain the desired effect, as noted in this quote.

“If I can be frank, I think some of the advertising and some of the material that is presented to people in the community - and I talked about this at the lead coalition - the literacy level is too high. It needs to be simpler. Remediation? What does that mean to most people? Even certification or qualify… so I think they look at this, and a lot of words… they are really not sure what they are talking about.”

Most of the participants stated that health care organizations and organizations offering social services had a responsibility to educate their clients about lead and lead poisoning prevention, and recommended collaborations between these two entities draw on the respective strengths of each partner. Participants acknowledged that, indeed, some organizations such as Women, Infants and Children (WIC), Early Head Start, Community Action Partnership, the Lancaster Lead Coalition, and hospitals were educating caregivers about lead and its detrimental effects. 

“As part of an Early Head Start program, we try to educate our families on the importance of lead. It is mandatory that we, – or it is required I should say – that we test each child in our program for lead. We test their lead levels and then we also encourage their entire household to be tested as well, especially if there is a positive result. And we do the education that comes along with that. Head Start has been in the community, and a strong pillar of the Lancaster community, for a long time and they also have the same requirement.”

### 3.2. Housing Status

Participants indicated that housing status has an impact on the lead poisoning rates, mainly because there is a housing shortage in Lancaster. This housing shortage diminishes tenants’ ability to request that their landlord make their home lead safe. Participants further noted that renters may be fearful and feel intimidated to approach, or make a complaint against, their landlord if they live in a home with lead paint. Several participants mentioned tenants’ concerns of eviction as the main source of fear.

“I think also Lancaster County has a lack of affordable housing and if somebody is able to find an affordable unit—they don’t want to—they feel so grateful that they don’t want to rock the boat, they don’t want to ask their landlord to do anymore because then they’re afraid the landlord will say ‘you’re a pain in the neck, I don’t want to rent to you, you know, I’m not going to renew your lease,’ which is not right and not fair.”

“I know Lancaster has an ordinance that requires landlords, when they are renting to children, to actually have a lead safe environment—if your landlord is offering a rent that is on the low side, and you don’t think you can afford anything more than you have, you are not going to report that landlord for not following that ordinance because you are going to be afraid of being evicted. Because, even though technically they can’t retaliate against you about reporting them, the fact is we all know that there is a lot of ways to retaliate against people. … So I think that there are many reasons why people will be afraid to report their landlords, and that is really a current form of discrimination in my mind, and it is environmental justice discrimination because it will be people who won’t report, people who are new immigrants, people who are illegal immigrants, people who just know they can’t afford anything more than what they have, and those are all taking advantage of vulnerabilities.”

A few participants were hopeful that the new lead ordinance, requiring landlords to make homes lead safe in order to lease out the home to a family with a child under the age of six years, will eliminate the fear on the part of tenants. A few participants did not think the lead safe requirement should be limited to families with children under the age of six, instead it should be extended to all homes in Lancaster. 

“I think that will make a difference, if the landlords are held accountable and they feel like ‘if I don’t do this there will be repercussions.’ I think that is really, really important.” 

However, some participants recommended that landlords perhaps need some educational sessions about lead and lead poisoning. They mentioned that most interventions are targeted towards the tenants and assume that landlords are knowledgeable about the effects of lead and choose not to abate their homes. They suggested conducting meetings with landlords to share educational material on the causes, effects, and prevention of lead poisoning. Additionally, participants voiced that educating tenants about their rights would increase their autonomy, and even suggested having a public record of addresses of properties at which a child was diagnosed with lead poisoning.

“I think it’s a lot of ongoing education to - not only for the people that we are trying to help with getting lead tested, the families that are - but also for the landlords and for the communities that are—because the landlords develop a community of their own, um, and I think it’s important to share that same information with them.”

“The flyers and the news are pushing out the ordinance, but they are not pushing ways to make it better, like how to educate the parents, so this ordinance is not so big a deal for these homeowners and these landlords.”

### 3.3. Competing Interests

Several respondents contributed narratives indicating that more pressing needs of community members are likely to be prioritized over lead poisoning and thereby reduce the attention and minimizing the actions necessary to reduce lead poisoning. Examples of such competing interests include: economic difficulties, housing status, work commitments, other illnesses, and time constraints. 

“Probably another one (barrier), is just the, um the difficult lives that some of our families are leading, and the priority of lead. They just may not always see it as the high priority that it probably should be. You know they have transportation problems; you know they have job problems; their family might be hungry; they have more pressing problems in their minds I think.” 

Competing needs, coupled with limited knowledge about the effects of lead poisoning, can cause community members to compromise their health. Additionally, participants shared that because lead poisoning is passive, and presents with symptoms that are similar to other illnesses, parents are less likely to give the same degree of attention to lead poisoning as they do other illnesses. 

“If you’ve got a parent that said ‘I’ve grown up with lead-based paint, my kids are fine.’ Being lead poisoned, there isn’t any obvious … there isn’t obvious signs, you know their face isn’t purple, their arm isn’t falling off, you know, okay they might be a little hyper, and yeah maybe they’re not as smart as their cousin, but, you know, they’re fine. So when you are in survival mode, and especially if you’re low income, getting through the day and getting your kids to work and keeping your job is number one. So unless something is in your face forcing you to deal with it, it’s very easy to put off, not that they don’t care, in no way want to imply that.” 

Furthermore, respondents acknowledged that the fact that lead poisoning has no immediate specific symptoms, unlike other illnesses in which a child may present with a fever or flu-like symptoms, exacerbates the priority of competing interests. Respondents did not provide direct recommendations to address the idea that lead poisoning ranks low on caregivers’ priority lists; however, some of the responses did offer implicit recommendations. It is plausible that having services related to lead poisoning prevention embedded in facilities or organizations where parents have to go to attend to the higher-ranking needs might inherently help parents to understand the adverse effects of lead poisoning. 

“So I think that an approach for physicians is to make the information about how to connect with the right people, either integrate it into the medical record or integrate it into something having to do with their automatic processes that they already go through, but something that makes it so easy that it’s impossible to not know… to overlook.”

## 4. Discussion

The results of this study identify low levels of knowledge about lead and lead poisoning, housing status, and competing interests as factors that contribute to the high rates of lead poisoning in Lancaster. The findings of this qualitative study are consistent with earlier quantitative studies. Mehta et al. administered the Chicago Lead Knowledge Test (CLKT) to over 2000 parents of children under the age of six years and the mean score was 50.8% (12.2/24) [15]. Similarly, another study demonstrated that only 15% of participants identified lead paint dust as a source of lead [16]. More recently, Bogar et al. also administered the CLKT to adolescents ages 13 to 18 and the mean score was 50% (12/24) [17]. The low knowledge levels in different regions over time suggests the need to design interventions that will effectively educate communities about lead. Both the study conducted by Bogar et al. and the study presented herein, were conducted after the lead crisis in Flint, Michigan, and yet, lead knowledge levels remain low. One would expect the Flint water crisis to initiate conversations around lead and thus increase awareness. 

Materials designed for health promotion can improve knowledge levels; however, for enhanced and sustained efficacy, it is important that such interventions are tailored to target specific age groups and their associated literacy level. The Centers for Disease Control and Prevention offers guidelines on how to prepare health promotion material and stresses the use of terms with which the audience can relate and material that is culturally sensitive [18]. Other researchers have created lead poisoning health promotion material in non-print form through videotapes [19] and public health media campaigns [20]. The efficacy of the videotape was assessed by asking participants to take a pre- and post-test of a shortened CLKT, which yielded significant results in favor of the tape. Given that majority of the population in Lancaster either has less than a high school diploma or had a high school diploma, health promotion materials on lead and lead poisoning prevention should be designed at an appropriate literacy level, ideally not higher than an eight-grade reading level [18].

Consistent with this study, several quantitative studies identify rental status as a predictor of EBLL [21,22,23]. In one study, the odds of renting a home and having an EBLL was 3.2 times the odds of owning a home and having an EBLL [22]. Children in families that rent property might be at an increased risk because of the landlord’s insufficient maintenance of the homes, coupled with the tenant’s unwillingness and/or powerlessness to make any home improvements. In addition, fear of eviction in a city with a housing shortage is a reality that might even impact the ability of renters to report peeling paint to their landlord. Professionals in Maryland have described this scenario as a challenge in addressing lead poisoning [24]. 

In November 2017, Lancaster City passed an ordinance [25] that requires landlords to present tenants with a lead safe certification, prepared by a lead-based paint risk assessor, prior to occupation of the rental unit to a family with a child under the age of six. Once signed by the tenant, the landlord is required to provide a copy of the signed certificate to the City Code Compliance and Inspection Office of Lancaster City. If landlords are discovered to violate the requirements of the ordinance when a child is diagnosed with an EBLL (EBLLs trigger a home inspection), tenants can abate rent until the dwelling is certified as lead safe at the cost of the landlord and/or may serve notice within 30 days of diagnosis and receive the security deposit refund at the time the premises are vacated. It is likely that, if well implemented, this ordinance could contribute to decreasing the rates of lead poisoning as this ordinance is more restrictive than the Environmental Protection Agency Real Estate Disclosure that requires that tenants and buyers receive a pamphlet titled “Protect your family from lead in your home”; be provided with information about lead-based paint or lead-based paint hazards in the home; and a statement that the landlord/seller has adhered to all lead notification requirements. Other cities have enacted regulations and policies to address lead poisoning, although it is still unclear if these regulatory endeavors have had an impact [26]. Strict enforcement of ordinances and rules such as the lead disclosure [8] might contribute to reducing lead exposures. However, the identification of fear suggests that either the ordinance should be enforced more strictly so the responsibility does not rest on the renters otherwise the renters need to be educated on the rights bestowed to them by the ordinance. 

While educating renters or tenants about the ordinance is of value, it is also beneficial to educate landlords on lead and lead poisoning. This is a feasible option as previous occupational health education provided to workers, who could potentially bring lead into their homes, [27,28] resulted in a change in attitude and increased practice in protective behaviors regarding lead poisoning. In a study by Phoenix et al. an educational session on lead hazards resulted in an increase in knowledge [29] for realtors. Landlords would likely benefit from an educational intervention focused on lead poisoning prevention. The intervention might result in landlords investing more resources in maintaining homes, applying for funds to make homes lead safe, and complying with the lead ordinance. Through the Lancaster Housing Opportunity Partnership, both landlords and renters can be informed about the ordinance. The existing programs such as the Renters Education Program should, in addition to providing tenants with tools to be successful renters, expose renters to the ordinance and their respective rights. Landlords participating in education about the hazards of lead, specifically to children under six, may be more likely to apply for the HUD funds or take their own initiative and make their homes lead safe because they realize how harmful it can be to their tenants’ child(ren).

Finally, this study identifies competing interests as a factor that potentially contributes to the high rates of lead poisoning. Tenants highly prioritize basic needs such as housing, transportation, and access to food over lead poisoning, and while health is important, most burdened families take a reactive approach as opposed to a proactive approach towards health [30,31]. This passive approach is detrimental to lead poisoned children because lead poisoning does not necessarily have unique or obvious symptoms. The irreversible nature of neurological damage from lead poisoning and the identified low lead knowledge levels compound the issue. Therefore, individuals, and families with children, at risk of lead poisoning need individualized interventions that place an emphasis on lead poisoning diagnosis, testing, and prevention incorporated into their existing lifestyles. In a study by Jordan et al. an already burdened family was more likely to adopt practices and behaviors that were easy to implement and did not add any steps to their daily routine [32]. Systematic and organizational level approaches might be more suitable to mitigate lead poisoning by developing collaborations, creating one-stop-shops for lead-related services, or streamlining processes to minimize steps and efforts to address lead poisoning. An example of this streamlined approach would be if a physician gave their patient’s family health promotion materials while simultaneously testing the child for lead poisoning and getting assistance with completing forms for lead abatement, in the event that child has an elevated blood lead level. Although it may be hard to reach the uninsured population through primary care physicians, health promotion materials can be disseminated through various avenues where families access social services including Early Head Start, free clinics, day care facilities, and faith-based communities.

In Lancaster, including a lead assessment clinic at Women Infants and Children (WIC), as was previously the case, can increase the rates of testing for lead poisoning. The results from this finger prick test were available in the same visit minimizing trips to the health care facility. Parents/Guardians of low-income families are less likely to be able to get time off for frequent health care visits and so initiatives to keep doctor visits to a minimum by including testing for lead poisoning in the wellness visits will address lead poisoning. Such initiatives require organizations like WIC or Head Start to prepare grant proposals for funding for relevant expertise.

### Limitations

This study acknowledges that using a convenience sample might have excluded participants who could have provided different perspectives; however, the study reached saturation. In addition, since this is a qualitative study, the findings might not be generalized to other populations and regions, however these data are valuable to Lancaster and highlight the need for other cities and/or counties to understand geographic-specific factors that might contribute to high rates of lead poisoning. 

## 5. Conclusions

Our findings share professionals’ perspectives on the issue of lead poisoning in Lancaster County and City, specifically that low knowledge levels, renting homes versus home ownership, and competing priorities contribute to the high rates of lead poisoning. These results will (i) guide public health practitioners in developing appropriate interventions that address the issue of childhood lead poisoning and (ii) create a database of information that can be utilized to conduct further research using quantitative studies.

## References

[1] Centers for Disease Control and Prevention. Lead. https://www.cdc.gov/nceh/lead/default.htm.

[2] Chisolm J., Harrison H. (1956). The Exposure of Children to Lead. J. Am. Acad. Pediatrics..

[3] Leung A.K., Hon K.L. (2016). Attention-deficit/hyperactivity disorder. Adv. Pediatr..

[4] Reuben A., Caspi A., Belsky D.W., Broadbent J., Harrington H., Sugden K., Houts R.M., Ramrakha S., Poulton R., Moffit T.E. (2017). Association of childhood blood lead levels with cognitive function and socioeconomic status at age 38 years and with iq change and socioeconomic mobility between childhood and adulthood. JAMA.

[5] Evens A., Hryhorczuk D., Lanphear B.P., Rankin K.M., Lewis D.A., Forst L., Rosenberg D. (2015). The impact of low-level lead toxicity on school performance among children in the Chicago Public Schools: A population-based retrospective cohort study. Environ. Health..

[6] Chen A., Cai B., Dietrich K.N., Radcliffe J., Rogan W.J. (2007). Lead exposure, IQ, and behavior in urban 5- to 7-year-olds: Does lead affect behavior only by lowering IQ?. Pediatrics.

[7] Stretesky P.B., Lynch M.J. (2001). The relationship between lead exposure and homicide. Arch. Pediatr. Adolesc. Med..

[8] U.S. Department of Housing and Urban Development. The Lead Disclosure Rule|HUD.gov/U.S. Department of Housing and Urban Development (HUD). https://www.hud.gov/program_offices/healthy_homes/enforcement/disclosure.

[9] State of Pennsylvania. *Act 39*; 2018; Vol. 742. https://www.legis.state.pa.us/cfdocs/legis/li/uconsCheck.cfm?yr=2018&sessInd=0&act=39.

[10] School District of Lancaster 2019 Lead Testing. https://www.lancaster.k12.pa.us/lead-testing/building-results/.

[11] U.S. Department of Housing and Urban Development (2016). Fiscal Year 2016 Lead Based Paint Hazard Control Grants.

[12] Pennsylvania Department of Health. D2016. 2015 Childhood Lead Surveillance Annual Report. https://www.health.pa.gov/topics/Documents/Environmental%20Health/2015%20Lead%20Surveillance%20Annual%20Report.pdf.

[13] Pennsylvania Department of Health. December 2018. 2017 Childhood Lead Surveillance Annual Report. https://www.health.pa.gov/topics/Documents/Environmental%20Health/2017%20Childhood%20Lead%20Surveillance%20Annual%20Report.pdf.

[14] (2018). United States Census Bureau. https://www.census.gov/quickfacts/fact/table/lancastercountypennsylvania/PST040218.

[15] Mehta S., Binns H.J. (1998). What do parents know about lead poisoning? The Chicago Lead Knowledge Test. Pediatric Practice Research Group. Arch. Pediatr. Adolesc. Med..

[16] Mahon I. (1997). Caregivers’ knowledge and perceptions or preventing childhood lead poisoning. Public Health Nurs..

[17] Bogar S., Szabo A., Woodruff S., Johnson S. (2017). Urban youth knowledge and attitudes regarding lead poisoning. J. Community. Health..

[18] (2010). Centers for Disease Control and Prevention. Simply Put. 3rd edition. https://www.cdc.gov/healthliteracy/pdf/Simply_Put.pdf.

[19] Kersten H.B., Moughan B., Moran M.M., Spector N.D., Smals L.E., DeLago C.W. (2004). A videotape to improve parental knowledge of lead poisoning. Ambul. Pediatr..

[20] Greene D., Tehranifar P., DeMartini D.P., Faciano A., Nagin D. (2015). Peeling lead paint turns into poisonous dust. Guess where is ends up? A media campaign to prevent childhood lead poisoning in New York City. Health Educ. Behav..

[21] Lanphear B.P., Byrd R.S., Auinger P., Schaffer S.J. (1998). Community characteristics associated with elevated blood lead levels in children. Pediatrics.

[22] Lanphear B.P., Hornung R., Ho M. (2005). Screening housing to prevent lead toxicity in children. Public Health Rep..

[23] Jacobs D.E., Clickner R.P., Zhou J.Y., Viet S.M., Marker D.A., Rogers J.W., Zeldin D.C., Friedman W. (2002). The prevalence of lead based paint hazards in U.S. housing. Environ. Health Perspect..

[24] Beard B., DeCaprio A., Hoffman A., Leiter-Mason E. 2015. Taking the Lead on Lead Poisoning, Maryland Governor’s Summer Internship Program. https://shrivercenter.umbc.edu/files/2015/11/2015-Lead-Poisoning.pdf.

[25] Lead Ordinance Lancaster (2017). Administrative Ordinance No 14-2017.

[26] Korfmacher K.S. (2010). Boundary networks and Rochester’s “smart”lead law: The use of multidisciplinary information in a collaborative policy process. New Solut..

[27] Lormphong S., Morioka I., Miyai N., Yamamoto H., Chaikittiporn C., Thiramanus T., Miyashita K. (2004). Occupational health education and collaboration for reducing the risk of lead poisoning of workers in battery manufacturing plant in Thailand. Ind. Health.

[28] Buzzetti A.J., Greene F., Needham D. (2005). Impact of a lead-safe training program on workers conducting renovation, painting and maintenance activities. Public Health Rep..

[29] Phoenix J.A., Green R.D., Thompson A.M. (2013). Can realtor education reduce lead exposure for vulnerable populations?. J. Environ. Health..

[30] Ross J.S., Bernheim S.M., Bradley E.H., Teng H.M., Gallo W.T. (2007). Use of preventive care by the working poor in the United States. Prev Med..

[31] Brunner-Ziegler S., Rieder A., Stein K.V., Koppensteiner R., Hoffmann K., Dorner T.E. (2013). Predictors of participation in preventive health examinations in Austria. BMC Public Health.

[32] Jordan C.M., Lee P.A., Oikon R., Pirie P.L. (2007). Messages from moms: Barriers to and facilitators of behavior change in a lead poisoning preventive education project. J. Health Commun..

